# Exposure to, and searching for, information about suicide and self-harm on the Internet: Prevalence and predictors in a population based cohort of young adults

**DOI:** 10.1016/j.jad.2015.06.001

**Published:** 2015-10-01

**Authors:** Becky Mars, Jon Heron, Lucy Biddle, Jenny L. Donovan, Rachel Holley, Martyn Piper, John Potokar, Clare Wyllie, David Gunnell

**Affiliations:** a School of Social and Community Medicine, University of Bristol, Oakfield House, Bristol BS8 2BN, United Kingdom; b Samaritans, United Kingdom; c PAPYRUS Prevention of Young Suicide, United Kingdom

**Keywords:** ALSPAC, Internet, Self-harm, Suicide attempt, Suicide

## Abstract

**Background:**

There is concern over the potential impact of the Internet on self-harm and suicidal behaviour, particularly in young people. However, little is known about the prevalence and patterns of suicide/self-harm related Internet use in the general population.

**Methods:**

Cross sectional study of 3946 of the 8525 participants in the Avon Longitudinal Study of Parents and Children (ALSPAC) who were sent a self-report questionnaire including questions on suicide/self-harm related Internet use and self-harm history at age 21 years.

**Results:**

Suicide/self-harm related Internet use was reported by 22.5% (886/3946) of participants; 11.9% (470/3946) had come across sites/chatrooms discussing self-harm or suicide, 8.2% (323/3946) had searched for information about self-harm, 7.5% (296/3946) had searched for information about suicide and 9.1% (357/3946) had used the Internet to discuss self-harm or suicidal feelings. Suicide/self-harm related Internet use was particularly prevalent amongst those who had harmed with suicidal intent (70%, 174/248), and was strongly associated with the presence of suicidal thoughts, suicidal plans, and history of self-harm. Sites offering help, advice, or support were accessed by a larger proportion of the sample (8.2%, 323/3946) than sites offering information on how to hurt or kill yourself (3.1%, 123/3946). Most individuals (81%) who had accessed these potentially harmful sites had also accessed help sites.

**Limitations:**

(i) There were differences between questionnaire responders and non-responders which could lead to selection bias and (ii) the data were cross-sectional, and we cannot conclude that associations are causal.

**Conclusions:**

Suicide/self-harm related Internet use is common amongst young adults, particularly amongst those with suicidal thoughts and behaviour. Both harmful and helpful sites were accessed, highlighting that the Internet presents potential risks but also offers opportunities for suicide prevention.

## Highlights

• We examined suicide/self-harm related Internet use in a sample of young adults.
• 22.5% of participants reported suicide/self-harm related Internet use.
• It was strongly associated with self-harm, suicidal thoughts, and suicidal plans.
• A greater proportion had accessed helpful sites than potentially harmful sites.
• The Internet presents potential risks but also offers opportunities for prevention.

## 1 Introduction

The potential impact of the Internet on self-harm and suicidal behaviour has been highlighted both as a public health concern and as an opportunity for prevention (Boyce, 2010), but little is known about the prevalence and patterns of suicide/self-harm (S/Sh) related Internet use in the general population. The Internet can provide a supportive environment in which to seek information and advice about self-harm/suicidal feelings and can help to reduce feelings of loneliness and isolation (Baker and Fortune, 2008; Baker and Lewis, 2013; Eichenberg, 2008; Harris and Roberts, 2013; Jones et al., 2011; Shaw and Gant, 2002; Whitlock et al., 2006). However, there is also concern that exposure to online S/Sh-related content may increase suicide risk amongst vulnerable individuals (Baker and Lewis, 2013; Harris and Roberts, 2013; Lewis and Baker, 2011; Lewis et al., 2012; Whitlock et al., 2006). Websites that encourage or facilitate suicide and sites containing technical information on suicide methods are easily accessed online (Biddle et al., 2008; Recupero et al., 2008; Sakarya et al., 2013) and S/Sh-related Internet use had been reported both in coroners’ records and by survivors of suicide attempts (Becker et al., 2004; Biddle et al., 2012; Gunnell et al., 2012; Prior, 2004). There is also concern that the ease at which information is shared online may contribute to the uptake of new suicide methods (Gunnell et al., 2014).

Despite these concerns, empirical data on S/Sh-related Internet use in the general population is lacking. Existing studies have typically analysed the content of forum posts, or conducted e-surveys with individuals responding to online adverts (Baker and Lewis, 2013; Eichenberg, 2008; Harris et al., 2009; Jones et al., 2011; Whitlock et al., 2006). For example, Harris et al. (2009) investigated Internet use amongst a sample of 290 adults who responded to an online survey and were considered to be at risk of suicide. Those who had used the Internet for suicide-related purposes were more likely to be unemployed, to live alone, to report psychiatric disorder and to have lower levels of education. A range of different sites were accessed including forums, suicide prevention sites, sites encouraging suicide, and suicide pact sites. However, the findings were based on data from a self-selecting high-risk sample and may not generalise to the wider population. Mitchell et al. (2014) investigated exposure to websites that encourage self-harm or suicide in a telephone survey of over 1500 Internet-using adolescents. Access to websites that encouraged self-harm or suicide was reported by 1% and was strongly associated with suicidal thoughts. The study did not ask about actual self-harm behaviours or investigate access to other sites such as help sites. In a sample of over 3500 school pupils, O’Connor et al. (2014) found that 18% of those who had self-harmed indicated the Internet or social networking sites influenced their decision to engage in self-harm.

Knowledge of S/Sh-related Internet use in the general population is important in order to gain a better understanding of the potential contribution of the Internet to suicide and self-harm behaviours, and to inform research, policy, and the development of online suicide prevention strategies. The aim of the present study was to investigate S/Sh-related Internet use amongst young adults in the community. Throughout the paper, we use the term self-harm to refer to individuals who have harmed with or without suicidal intent.

## 2 Methods

### 2.1 Sample

The Avon Longitudinal Study of Parents and Children (ALSPAC) is an ongoing population-based birth cohort study examining influences on health and development across the life-course. The ALSPAC core enroled sample consists of 14,541 pregnant women resident in the former county of Avon in South West England (United Kingdom), with expected delivery dates between 1 April 1991 and 31 December 1992 (Boyd et al., 2013). Of the 14,062 live births, 13,798 were singletons/first-born of twins and were alive at one year of age. Participants have been followed-up regularly since recruitment through questionnaires and research clinics (see study website: http://www.bristol.ac.uk/alspac which includes a fully searchable data-dictionary of available data http://www.bris.ac.uk/alspac/researchers/data-access/data-dictionary). Ethical approval for the study was obtained from the ALSPAC Law and Ethics committee and local research ethics committees. Written informed consent was obtained after the procedure(s) had been fully explained.

Questions on S/Sh-related Internet use and history of self-harm were included as part of a broader self-completion questionnaire, sent to study participants when they were aged 21 years (mean age 20.9 years). The questionnaire was sent to 8525 participants, of whom 4110 (48.2%) responded and 3946 (46.3%) provided data on their S/Sh-related Internet use and previous self-harm. Those who returned the questionnaire were more likely than non-respondents to be female, white, have lower birth order, a mother with higher education (assessed during pregnancy), and a higher parental social class (assessed during pregnancy). There was little evidence to suggest differences in mental health (assessed at age 18 years) (Supplementary Table 1).

### 2.2 Suicide/self-harm-related Internet use

Participants were asked four questions about their Internet use: (i) “A number of sites and chatrooms on the Internet discuss self-harm and suicide. Have you ever come across any of these sites?” (ii) “Have you ever looked for information about self-harm using a search engine (Google, Yahoo etc.)?”, (iii) “Have you ever looked for information about suicide using a search engine (Google, Yahoo etc.)?” and (iv) “Have you ever used the Internet to discuss self-harm or suicidal feelings with others (e.g. social networking sites, chatrooms, message boards, help sites)?”. Participants were asked not to include searches that were done only for an assignment or in relation to helping a friend/family member. Participants who responded positively to one or more of these four questions were classified as having S/Sh-related Internet use. The term S/Sh-related Internet use is used throughout the paper for simplicity, however, we recognise that this refers to a variety of different types of Internet use.

Participants who indicated that they had come across Internet sites that discuss self-harm or suicide were asked to select which sites they had read from a checklist (response options included the following: news reports about people who have hurt or killed themselves/personal accounts of people who have hurt themselves/general information about self-harm or suicide/sites dedicated to those who self-harm/sites offering advice, help, or support regarding self-harm or suicidal feelings/sites giving information about how to hurt or kill yourself). The paper focuses on the latter two response options, which we refer to as “helpful” and “potentially harmful” sites.

### 2.3 Self-harm and suicidal thoughts

The self-harm questions asked at age 21 years were based on those used in the Child and Adolescent Self-harm in Europe (CASE) study (Madge et al., 2008). Participants who responded positively to the item “have you ever hurt yourself on purpose in any way (e.g. by taking an overdose of pills or by cutting yourself)?” were classified as having a history of self-harm. A number of additional questions were then asked regarding the recency of self-harm, past year frequency, motivations for self-harm on the most recent occasion, lifetime history of suicidal self-harm and help-seeking. Participants were also asked about suicidal thoughts and plans.

#### 2.3.1 Suicidal self-harm

Participants were classified as having harmed with suicidal intent if (i) they selected “I wanted to die” as a response option to the question “Do any of the following reasons help to explain why you hurt yourself on that (i.e. the most recent) occasion?” or (ii) they responded “yes” to the question “On any of the occasions when you have hurt yourself on purpose, have you ever seriously wanted to kill yourself?”.

#### 2.3.2 Suicidal thoughts and plans

lifetime history of suicidal thoughts and plans were assessed with the questions “have you ever thought of killing yourself, even if you would not really do it” and “have you ever made plans to kill yourself”.

#### 2.3.3 Help-seeking

Participants were classified as having sought professional help for self-harm if they indicated they had sought medical help from a GP or hospital emergency department following their most recent act of self-harm, or had ever sought professional (counsellor/therapist, GP/doctor, psychologist/psychiatrist, mental health services/other mental health professional) help for self-harm or suicidal thoughts.

#### 2.3.4 Other variables considered

We also examined associations of S/Sh-related Internet use with: ethnicity; parent social class (professional/managerial or other occupations, the highest of maternal or paternal social class was used); Not in Education, Employment, or Training (NEET), assessed by self-report at age 21 years; number of friends seen regularly, assessed by self-report at age 21 years (<3 vs. 4 or more); depressive and anxiety disorder, assessed at a clinic held at 18 years using the computerised version of the Clinical Interview Schedule-Revised (CIS-R) (Lewis et al., 1992); and previous exposure to self-harm in friends and family, assessed by self-report at age 16 years.

#### 2.3.5 Statistical analyses

Cross tabulation and univariable logistic regression models were used to examine differences in the pattern of S/Sh-related Internet use according to gender, self-harm, and self-harm with and without suicidal intent. Associations with exposure variables and S/Sh-related Internet use were examined in (i) the whole sample and (ii) the subsample who had self-harmed. Associations were also examined separately for helpful sites (offering help, advice, or support) and potentially harmful sites (offering information on how to hurt or kill yourself).

We investigated the possible influence of selective participation (non-response to questionnaires) on our estimates using a combination of multiple imputation and inverse probability weighting (MI/IPW) (Seaman and White, 2014; Seaman et al., 2012). All analyses were conducted using Stata version 13.

## 3 Results

### 3.1 Prevalence and patterns of suicide/self-harm related Internet use in the total sample

Out of 3946 participants who provided data on their S/Sh-related internet use and previous self-harm 22.5% (95% CI 21.1% to 23.8%, *n*=886) reported S/Sh-related Internet use. Altogether 11.9% (95% CI 10.9% to 13.0%, *n*=470) participants had come across Internet sites and chat rooms that discuss self-harm and suicide (Table 1); 8.2% (95% CI 7.4% to 9.1%, *n*=323) had looked for information about self-harm using a search engine; 7.5% (95% CI 6.7% to 8.4%, *n*=296) had looked for information about suicide using a search engine and 9.1% (95% CI 9.0% to 9.2%, *n*=357) had used the Internet to discuss self-harm or suicidal feelings. A greater proportion of individuals had accessed sites offering help, advice, or support (8.2%, 95% CI 7.4% to 9.1%, *n*=323) than had accessed sites offering information on how to hurt or kill yourself (3.1%, 95% CI 2.6% to 3.7%, *n*=123). Most (81%, *n*=100/123) of the individuals who had accessed potentially harmful sites had also accessed help sites. Females were more likely to report each of the four types of S/Sh-related Internet use than males. Similar prevalence estimates of S/Sh-related Internet use were obtained from the MI/IPW analyses (Supplementary Table 2).

### 3.2 Factors associated with suicide/self-harm related Internet use in the total sample

Table 2 describes the prevalence of characteristics associated with S/Sh-related Internet use in the total sample, and in the subsamples with and without Internet use. Those with S/Sh-related Internet use were substantially more likely to have a history of suicidal thoughts, plans or self-harm, and to have sought professional help, than those without [odds ratios (OR) 5.81 for suicidal thoughts, 11.7 for suicidal plans, 5.97 for self-harm and 9.40 for professional help-seeking]. Individuals who reported S/Sh-related Internet use were also more likely to have had depression or an anxiety disorder at age 18 years, to report knowing at age 16 years a friend or family member who had self-harmed and to have fewer than three close friends (Table 2). Associations with S/Sh-related Internet use were similar in the MI/IPW analyses (Supplementary Table 3).

When examining helpful sites and potentially harmful sites individually, the associations were broadly similar (available on request). Whilst those reporting suicidal thoughts and plans were at particular risk of accessing potentially harmful sites (11.1%, (*n*=85/765) of those with thoughts and 28.9% (*n*=52/180) of those with plans), these groups were also more likely to access help sites (23.9% (*n*=183/765) of those with thoughts and 42.8% (*n*=77/180) of those with plans).

### 3.3 Prevalence and patterns of suicide/self-harm related Internet use amongst those with a history of self-harm

Table 3 shows the pattern of S/Sh-related Internet use according to self-harm history. Altogether 20.8% (*n*=819/3946) young people in the sample had a history of self-harm. Just over half (51.3%, *n*=419/819) of those who had self-harmed reported S/Sh-related Internet use compared to 14.9% (*n*=467/3127) amongst those who had never self-harmed. Participants who had self-harmed were more likely to report each of the four types of S/Sh-related Internet use, particularly “looking for information about self-harm using a search engine” [OR=17.7, 95% CI 13.4 to 23.2]. A quarter (24.2%, *n*=198/819) of individuals who had self-harmed had read sites offering help, advice, or support; fewer (9.7%, *n*=79/819) had read sites providing information on how to hurt/kill yourself.

### 3.4 Factors associated with suicide/self-harm related Internet use amongst those with a history of self-harm

Similar factors were associated with S/Sh-related Internet use in the subsample who had self-harmed, as found for the whole sample (suicidal thoughts, suicidal plans, having sought professional help, depression and anxiety disorder). There was little evidence of an association with gender, number of close friends, or exposure to self-harm in friends/family (Supplementary Table 4).

### 3.5 Differences in prevalence and patterns of suicide/self-harm related Internet use between those who had harmed with and without suicidal intent

Table 4 shows the pattern S/Sh-related Internet use amongst those who had self-harmed with and without suicidal intent. Information about suicidal intent was available for 97.1% (*n*=795/819) of those who had self-harmed. Out of the total available sample (3922), 14.0% (*n*=547) had harmed without suicidal intent and 6.3% (*n*=248) had harmed with suicidal intent on at least one occasion. The proportion of young people reporting S/Sh-related Internet use was higher amongst those with a history of suicidal self-harm than amongst those with non-suicidal self-harm (70.2% (*n*=174/248) vs. 42.1% (*n*=230/547), OR=3.24, 95% CI 2.35 to 4.47) (Table 4). Almost half of those with suicidal self-harm had searched for information about suicide (48.0%, *n*=119/248), and self-harm (49.2%, *n*=122/248), and 30.2% (75/248) had used the Internet to discuss suicidal feelings. This compares with 11.7% (64/547), 21.9% (120/547), and 14.3% (78/547) respectively amongst those with non-suicidal self-harm. Those with suicidal self-harm were more likely than those with non-suicidal self-harm to have accessed each of the different types of site (OR range 2.51–5.19), including sites offering help, advice, or support (39.9% (*n*=99/248) vs. 17.4% (*n*=95/547)) and sites providing information on how to hurt/kill yourself (20.6% (*n*=51/248) vs. 4.7% (*n*=26/547)).

## 4 Discussion

We found high levels of suicide/self-harm -related Internet use amongst young adults in the community. Almost a quarter of the sample (886/3946) had come across a site that discussed self-harm or suicide, looked for information about self-harm or suicide using a search engine, or used the Internet to discuss self-harm or suicidal feelings. S/Sh-related Internet use was particularly prevalent amongst those who had harmed with suicidal intent (70%). Sites offering information on how to hurt or kill yourself were accessed by a smaller proportion of individuals than sites offering help, advice or support. Access to these potentially harmful sites was increased amongst young adults with self-harm, suicidal thoughts, and suicidal plans (range 10–29%); however our findings suggest that most individuals had also accessed help sites.

### 4.1 Findings in relation to existing literature

The prevalence of self-harm in ALSPAC is somewhat higher than other population-based studies that have investigated self-harm (Evans et al., 2005; Hawton et al., 2002). This has been discussed in a previous paper (Kidger et al., 2012) and is likely due to methodological differences in sample, setting and the question we used. Our estimate of suicidal self-harm is broadly comparable with other population studies of young adults (range 5.4–8.8%) (Fergusson et al., 2005; Goldman-Mellor et al., 2014).

While Internet search studies have highlighted that pro-suicide information and details of suicide methods are easily accessible online (Biddle et al., 2008; Recupero et al., 2008; Sakarya et al., 2013), such studies do not tell us anything about the actual number of individuals who access such sites. In the youth Internet safety survey, Mitchell et al. (2014) found that 1% of adolescents (aged 10–17 years) in the USA had accessed websites which encouraged self-harm or suicide. This compares with 3% in our sample of young adults. Similar to our findings, the risk of accessing these potentially harmful sites was considerably elevated amongst those with suicidal thoughts. We extend this work by examining other types of S/Sh-related Internet sites, such as help-sites, and by examining associations with characteristics including self-harm and suicidal thoughts.

A recent systematic review of 14 studies concluded that the Internet has both positive and negative effects on young people at risk of self-harm or suicide (Daine et al., 2013). However, a few studies of S/Sh-related Internet use have distinguished between helpful and potentially harmful online content. In a content analyses of suicide-related websites, Till et al. (2014) found protective website characteristics outweighed harmful characteristics by 2:1. This is consistent with our finding that a greater proportion of young adults had accessed sites offering help, advice, or support than sites offering information on how to hurt or kill yourself. The factors associated with access to these two types of site were similar, which is not surprising, given the high level of overlap between them. This overlap could also partly reflect difficulties in classifying sites as either “helpful” or “harmful”, as some offer concurrent suicide-promoting and help-promoting content (Till and Niederkrotenthaler, 2014).

There is concern that individuals may turn to the Internet as an alternative to seeking professional help. Our findings suggest that those who report S/Sh-related Internet use were actually more likely to have sought professional help for self-harm/suicidal thoughts than those without. This could reflect the strong associations found between S/Sh-related Internet use and psychopathology, which in turn is related to help-seeking (Biddle et al., 2004), however, associations did not attenuate when we controlled for depression and anxiety disorder (results available on request). Harris et al. (2009) also investigated help-seeking and in contrast to our study, they found similar levels of mental health professional contact between those with and without suicide-related Internet use. The difference in findings is possibly due to differences in methodology; whereas we examined Internet use in a population sample of young adults, participants in the Harris et al. sample were older (~60% were over 25 years) and were selected to be at high risk for suicide.

Females were more likely to report S/Sh-related Internet use than males in our study. However this finding is likely due to a greater proportion of females having a history of self-harm, which was found to be strongly associated with S/Sh-related Internet use. There was little evidence to suggest gender differences amongst the subsample who had self-harmed. Those who reported S/Sh-related Internet use were also more likely to have psychopathology, including suicidal thoughts and plans. It is possible that individuals who are suicidal are using the Internet to learn more about suicide/self-harm, to research potential suicide methods, or to look for help or advice.

Qualitative research is needed to explore the reasons why individuals access S/Sh-related information online, particularly for the subgroup without self-harm, suicidal thoughts or plans (38% of those with S/Sh-related Internet use did not have self-harm, suicidal thoughts or plans). Those who reported S/Sh-related Internet use were more likely to have exposure to self-harm in friends/family, and so it is possible that some individuals were using the Internet to look for information on another’s behalf. However, participants were instructed not to include searches conduced in relation to helping a friend/family member. Those without self-harm who reported S/Sh-related Internet use also had higher rates of suicidal thoughts and plans and higher psychopathology than those without (Supplementary Table 5), suggesting that S/Sh-related Internet use is a marker of risk, even amongst those who have no prior self-harm history. It is also possible that access to S/Sh-related content might deter some individuals from engaging in self-harm.

### 4.2 Strengths and weaknesses

Despite widespread concerns about the potential for the Internet to promote or facilitate suicide, there is a lack of empirical data on S/Sh-related Internet use in the population. Existing studies have largely been based on highly self-selecting samples of Internet users responding to online surveys (Baker and Lewis, 2013; Eichenberg, 2008; Harris et al., 2009; Jones et al., 2011; Whitlock et al., 2006). As far as we are aware, this is the first study to provide estimates of suicide/self-harm related Internet use in the community, including prevalence estimates of use of search engines and interactive forums to research and discuss suicide/self-harm. Moreover, we examined patterns of use in a large population-based sample of young adults; this is an important age-group to investigate given their high levels of Internet use, and of social media in particular (Durkee et al., 2011). We were also able to compare characteristics of those with and without S/Sh-related Internet use, and examine associations separately for those with and without a history of self-harm.

The findings need however to be interpreted in light of several limitations. First, those who responded to the age 21 year questionnaire differed to non-responders on a range of characteristics (gender, ethnicity, birth order, maternal education and social class), and this non-random response may have led to selection bias. We used a combination of multiple imputation and inverse-probability weighting to examine the possible impact of missing data on our estimates and findings were very similar, suggesting that sample attrition did not have a substantial impact on the prevalence of S/Sh-related Internet use. Second, the data were cross-sectional, and therefore it was not possible to examine how S/Sh-related Internet use may influence future self-harm behaviour (Dunlop et al., 2011; Sueki, 2013; Sueki et al., 2014). Third, the reference period for Internet use and self-harm was ‘lifetime’ and reporting may be subject to recall bias or may be influenced by current mood state. Fourth, our definition of S/Sh-related Internet use was broad, comprising both self-harm and suicide-related content, and included those who had come across relevant sites, used a search engine, or had taken part in an online discussion. Although we estimated the prevalence separately for each type of Internet use, we did not undertake a detailed investigation of the risk factors associated with each. Finally, we do not know any additional information about the motivations surrounding S/Sh-related Internet use, the nature of the searches conducted, how individuals prioritise the content they come across, and how they use it, or the specific sites accessed. It is also unclear how sites are interpreted and evaluated by users, and what constitutes “helpful” content. We are addressing some of these issues in an ongoing qualitative study of ALSPAC members [Biddle et al., Department of Health grant reference PRP 023/0163].

### 4.3 Clinical and public health implications

In this sample of young adults, high levels of S/Sh-related Internet use were reported, particularly amongst those with self-harm, suicidal thoughts, and plans. This underscores the importance of the Internet in public health approaches to suicide prevention. Policy makers should work closely with Internet service providers to remove or restrict access to sites that actively promote suicide or self-harm, and help vulnerable individuals connect with helpful sites offering effective advice and support tailored towards young people. We found a greater proportion of individuals in this study had accessed helpful sites than potentially harmful sites. While reassuring, further work is needed to understand how content is interpreted, and the benefits and harms associated with different types of site use. Our findings also suggest that S/Sh-related Internet use is a potentially useful marker of risk, and could be incorporated into clinical assessments of individuals presenting with self-harm or suicidal thoughts as an additional indicator of severity.

## Role of funding source

The UK Medical Research Council and the Wellcome Trust (Grant ref: 092731) and the University of Bristol provide core support for ALSPAC. All material presented in this publication was drawn from independent research commissioned and funded by the Department of Health Policy Research Programme (“Exploring the Use of the Internet In Relation To Suicidal Behaviour: Identifying Priorities for Prevention”, PRP 023/0163). The views expressed in this publication are those of the author(s) and not necessarily those of the Department of Health.

*This publication is the work of the authors who serve as guarantors for the contents of this paper. The study sponsor had no further role in the study design and collection, analysis and interpretation of data or in the writing of the article and the decision to submit it for publication.*

*DG and JLD are National Institute for Health Research Senior Investigators.*

## Conflict of interest

None of the authors report any conflict of interest.

## Figures and Tables

**Table 1:** Proportions of male and female participants who had (a) encountered sites with suicide/self-harm related content, or (b) searched the Internet for information about suicide/self-harm, or (c) discussed self-harm or suicidal feelings using the Internet, and the types of sites they encountered.

Suicide/self-harm related Internet use, age 21 years	Total sample *N*=3946	Males *N*=1536	Females *N*=2410	*P* value⁎
**Any suicide/self-harm related Internet use**	886 (22.5%)	309 (20.1%)	577 (23.9%)	0.005
*Seen sites/chatrooms discussing suicide/self-harm*	470 (11.9%)	159 (10.4%)	311 (12.9%)	0.016
*Looked for information about self-harm using search engine*	323 (8.2%)	81 (5.3%)	242 (10.0%)	<0.001
*Looked for information about suicide using search engine*	296 (7.5%)	99 (6.5%)	197 (8.2%)	0.044
*Used Internet to discuss self-harm or suicidal feelings*	357 (9.1%)	118 (7.7%)	239 (9.9%)	0.017
				
**Type of site**				
News reports about people who have hurt or killed themselves	427 (10.8%)	144 (9.4%)	283 (11.7%)	0.020
Personal accounts of people who have hurt themselves	358 (9.1%)	121 (7.9%)	237 (9.8%)	0.037
General information about self-harm or suicide	402 (10.2%)	129 (8.4%)	273 (11.3%)	0.003
Sites dedicated to those who self-harm	175 (4.4%)	44 (2.9%)	131 (5.4%)	<0.001
Sites offering help, advice, or support	323 (8.2%)	86 (5.6%)	237 (9.8%)	<0.001
Information on how to hurt or kill yourself	123 (3.1%)	36 (2.3%)	87 (3.6%)	0.026

⁎ Difference between males and females.

**Table 2:** Factors associated with suicide/self-harm related Internet use (*n*=3,946).

	Total sample *N*=3,946	No suicide/self-harm related Internet use *n*=3,060	Suicide/self-harm related Internet use *n*=886	OR [95% CI]	P value⁎
Gender, % female	61.1% (2,410/3,946)	59.9% (1,833/3,060)	65.1% (577/886)	1.24 [1.07 to 1.46]	0.005
Ethnicity, % non white	4.0% (150/3787)	3.8% (110/2,933)	4.7% (40/854)	1.26 [0.87 to 1.83]	0.219
Social class					
*Professional/managerial*	64.9% (2,398/3,695)	64.7% (1,859/2,872)	65.5% (539/823)	0.97 [0.82 to 1.14]	0.686
*other*	35.1% (1,297/3,695)	35.3% (1,013/2,872)	34.5% (284/823)		
Suicidal thoughts, age 21 years	19.5% (765/3,925)	12.2% (371/3,043)	44.7% (394/882)	5.81 [4.90 to 6.90]	<0.001
Suicidal plans, age 21 years	4.6% (180/3,924)	1.5% (46/3,044)	15.2% (134/880)	11.7 [8.30 to 16.5]	<0.001
Self-harm, age 21 years	20.8% (819/3,946)	13.1% (400/3,060)	47.3% (419/886)	5.97 [5.04 to 7.07]	<0.001
Sought professional help for self-harm or suicidal thoughts, age 21 years	5.6% (221/3,946)	2.2% (67/3,060)	17.4% (154/886)	9.40 [6.98 to 12.7]	<0.001
Self-harm exposure (family/friend), age 16 years	44.1% (1,328/3,010)	40.3% (938/2,327)	57.1% (390/683)	1.97 [1.66 to 2.34]	<0.001
Depression, age 18 years	7.3% (186/2,561)	5.2% (104/1,987)	14.3% (82/574)	3.01 [2.22 to 4.10]	<0.001
Anxiety, age 18 years	11.1% (284/2,561)	8.7% (172/1,987)	19.5% (112/574)	2.56 [1.97 to 3.31]	<0.001
					
Number of close friends, age 21 years					
*3+ friends*	96.8% (3,674/3,795)	97.4% (2,860/2,936)	94.8% (814/859)	2.08 [1.43 to 3.03]	<0.001
*0-2 friends*	3.2% (121/3,795)	2.6% (76/2,936)	5.2% (45/859)		
Not in employment, education, or training (NEET) age 21 years	8.2% (311/3,785)	7.9% (232/2,928)	9.2% (79/857)	1.17 [0.90 to 1.54]	0.225

Number of participants with no suicide/self-harm related internet use 3,060; number of participants with suicide/self-harm related Internet use 886

⁎ Difference between those with and without suicide/self-harm related internet use

**Table 3:** Pattern of suicide/self-harm related Internet use according to self-harm history.

Suicide/self-harm related Internet use, age 21 years	No self-harm *N*=3127	Self-harm *N*=819	OR [95% CI]	*P* value ⁎
**Any suicide/self-harm related Internet use**	467 (14.9%)	419 (51.2%)	5.7 [5.04–7.06]	<0.001
*Seen sites/chatrooms discussing suicide/self-harm*	225 (7.2%)	245 (29.9%)	5.51 [4.50–6.74]	<0.001
*Looked for information about self-harm using search engine*	75 (2.4%)	248 (30.3%)	17.7 [13.4–23.2]	<0.001
*Looked for information about suicide using search engine*	108 (3.5%)	188 (23.0%)	8.33 [6.47–10.7]	<0.001
*Used Internet to discuss self-harm or suicidal feelings*	192 (6.1%)	165 (20.2%)	3.86 [3.09–4.83]	<0.001
				
**Type of site**				
News reports about people who have hurt or killed themselves	207 (6.6%)	220 (26.9%)	5.18 [4.20–6.39]	<0.001
Personal accounts of people who have hurt themselves	154 (4.9%)	204 (24.9%)	6.40 [5.11–8.03]	<0.001
General information about self-harm or suicide	182 (5.8%)	220 (26.9%)	5.94 [4.79–7.37]	<0.001
Sites dedicated to those who self-harm	54 (1.7%)	121 (14.8%)	9.87 [7.08–3.7]	<0.001
Sites offering help, advice, or support	125 (4.0%)	198 (24.2%)	7.66 [6.02–9.73]	<0.001
Information on how to hurt or kill yourself	44 (1.4%)	79 (9.7%)	7.48 [5.13–10.9]	<0.001

⁎ Difference between those with and without a history of self-harm.

**Table 4:** Pattern of suicide/self-harm related Internet amongst those who had self-harmed with and without suicidal intent.

Suicide/self-harm related Internet use, age 21 years	Non-suicidal self-harm *N*=547	Suicidal self-harm *N*=248	OR [95% CI]	*P* value ⁎
**Any suicide/self-harm related Internet use**	230 (42.1%)	174 (70.2%)	3.24 [2.35–4.47]	<0.001
*Seen sites/ chatrooms discussing suicde/self-harm*	125 (22.9%)	115 (46.4%)	2.92 [2.12–4.02]	<0.001
*Looked for information about self-harm using search engine*	120 (21.9%)	122 (49.2%)	3.45 [2.50–4.75]	<0.001
*Looked for information about suicide using search engine*	64 (11.7%)	119 (48.0%)	6.96 [4.85–9.98]	<0.001
*Used Internet to discuss self-harm or suicidal feelings*	78 (14.3%)	75 (30.2%)	2.61 [1.82–3.74]	<0.001
				
**Type of site**				
News reports about people who have hurt or killed themselves	116 (21.2%)	100 (40.3%)	2.51 [1.81–3.48]	<0.001
Personal accounts of people who have hurt themselves	100 (18.3%)	99 (39.9%)	2.97 [2.13–4.15]	<0.001
General information about self-harm or suicide	111 (20.3%)	105 (42.3%)	2.88 [2.09–4.00]	<0.001
Sites dedicated to those who self-harm	47 (8.6%)	71 (28.6%)	4.27 [2.84–6.41]	<0.001
Sites offering help, advice, or support	95 (17.4%)	99 (39.9%)	3.16 [2.26–4.43]	<0.001
Information on how to hurt or kill yourself	26 (4.7%)	51 (20.6%)	5.19 [3.15–8.55]	<0.001

⁎ Difference between those who had harmed themselves with and without suicidal intent.
