# Supplementary material for: Exposure to, and searching for, information about suicide and self-harm on the Internet: Prevalence and predictors in a population based cohort of young adults
Source: J Affect Disord. 2015 Oct 1;185:239–45. doi: 10.1016/j.jad.2015.06.001 (PMC4550475; doi:10.1016/j.jad.2015.06.001)
Supplement: Supplementary file 1 — Supplementary material [file mmc1.docx]

**Supplementary Table 1: Comparison of responders and non-responders to the questionnaire at 21 years by key demographic variables ***

| **Variable** | **Description** | ***N*** | **Questionnaire non-responders**  **(N=4,415)** | **Questionnaire responders**  **(N=4,110)** | **χ^2^** | ***P* value** |
| --- | --- | --- | --- | --- | --- | --- |
| Child gender | Male | 4,124 | 2,510 (56.9%) | 1,614 (39.3%) | 263.46 | <0.001 |
|  | Female | 4,401 | 1,905 (43.2%) | 2,496 (60.7%) |  |  |
| Birth order | First born | 3,738 | 1,803 (43.6%) | 1,935 (48.5%) | 24.01 | <0.001 |
|  | Second born | 2,879 | 1,497 (36.2%) | 1,382 (34.6%) |  |  |
|  | Third born plus | 1,514 | 838 (20.3%) | 676 (16.9%) |  |  |
| Maternal education | Degree | 1,212 | 422 (10.4%) | 790 (19.8%) | 248.25 | <0.001 |
|  | A level | 2,033 | 922 (22.8%) | 1,111 (27.8%) |  |  |
|  | O level | 2,789 | 1,460 (36.0%) | 1,329 (33.2%) |  |  |
|  | < O level | 2,020 | 1,249 (61.8%) | 771 (19.3%) |  |  |
| Parental social class | Professional/managerial | 4,468 | 1,974 (52.4%) | 2,494 (64.8%) | 120.87 | <0.001 |
|  | other | 3,149 | 1,794 (47.6%) | 1,355 (35.2%) |  |  |
| Child ethnicity | White | 7,531 | 3,742 (94.6%) | 3,789 (96.1%) | 9.41 | 0.002 |
|  | Non-white | 368 | 213 (5.4%) | 155 (3.9%) |  |  |
| Depression (age 18) | Yes | 300 | 105 (8.8%) | 195 (7.3%) | 2.39 | 0.122 |
|  | No | 3,563 | 1,094 (91.2%) | 2,469 (92.7%) |  |  |
| Anxiety (age 18) | Yes | 427 | 134 (11.2%) | 293 (11.0%) | 0.03 | 0.871 |
|  | No | 3,436 | 1,065 (88.8%) | 2,371 (89.0%) |  |  |
| Past year self-harm (age 18) | Yes | 351 | 96 (8.0%) | 255 (9.6%) | 2.45 | 0.117 |
|  | No | 3,512 | 1,103 (92.0%) | 2,409 (90.4%) |  |  |

**Comparison amongst those sent the questionnaire (N=8,525)*

*Information on mental health at 18 years is missing for over 50% of participants*

**Supplementary Table 2: Proportions of male and female participants who had a) encountered sites with suicide / self-harm related content, or b) searched the Internet for information about suicide / self-harm, or c) discussed self-harm or suicidal feelings using the Internet, and the types of sites they encountered: A comparison of original complete case and weighted analysis**

|  | Total sample | | Males | | Females | |
| --- | --- | --- | --- | --- | --- | --- |
|  | Original | Weighted | Original | Weighted | Original | Weighted |
| **Any suicide/self-harm related Internet use** | 22.5% | 23.3% | 20.1% | 20.3% | 23.9% | 26.0% |
| Seen sites/ chatrooms discussing suicide / self-harm | 11.9% | 12.3% | 10.4% | 10.5% | 12.9% | 13.9% |
| Looked for information about self-harm using search engine | 8.2% | 9.0% | 5.3% | 5.9% | 10.0% | 11.8% |
| Looked for information about suicide using search engine | 7.5% | 8.4% | 6.5% | 6.9% | 8.2% | 9.8% |
| Used Internet to discuss self-harm or suicidal feelings | 9.1% | 9.2% | 7.7% | 7.4% | 9.9% | 10.8% |
|  |  |  |  |  |  |  |
| **Type of site** |  |  |  |  |  |  |
| News reports about people who have hurt or killed themselves | 10.8% | 11.0% | 9.4% | 9.6% | 11.7% | 12.4% |
| Personal accounts of people who have hurt themselves | 9.1% | 9.5% | 7.9% | 8.2% | 9.8% | 10.6% |
| General information about self-harm or suicide | 10.2% | 10.7% | 8.4% | 9.0% | 11.3% | 12.2% |
| Sites dedicated to those who self-harm | 4.4% | 4.9% | 2.9% | 3.6% | 5.4% | 6.1% |
| Sites offering help, advice, or support | 8.2% | 8.4% | 5.6% | 5.8% | 9.8% | 10.7% |
| Information on how to hurt or kill yourself | 3.1% | 3.6% | 2.3% | 3.0% | 3.6% | 4.2% |

*We accounted for attrition using a combination of multiple imputation and inverse probability weighting (MI/IPW). IPW is an appropriate technique given the pattern of missing data in this study (as most data was taken from a single source most participants either had complete observed or completely missing information). Multivariable Imputation by Chained Equations was used as a preliminary step to impute missing values amongst the data used in the IPW missingness model. One hundred imputed datasets were generated for responders (n=3946) and for non- responders (n=4579). Imputations were generated separately for males and females to allow for possible gender differences. The datasets were pooled and a logistic regression model was derived to predict inclusion in the final sample (n=3946). A number of variables associated with non-response were included (e.g. indicators of socioeconomic adversity, maternal psychopathology, demographics, and mental health). The Hosmer-Lemeshow test indicated that the model provided a reasonable fit to the data. The inverse of the predicted probabilities from this model were then used to weight the subsequent analyses. The largest 1% of weights were truncated to the value of the 99^th^ percentile (10.26).*

**Supplementary Table 3: Factors associated with suicide/self-harm related Internet use: A comparison of original complete case and weighted analysis**

|  | No suicide/self-harm related Internet use | | Suicide/self-harm related Internet use | | OR [95% CI] | |
| --- | --- | --- | --- | --- | --- | --- |
|  | Original | Weighted | Original | Weighted | Original | Weighted |
| Gender, % female | 59.9% | 50.4% | 65.1% | 58.2% | 1.24 [1.07 to 1.46] | 1.37 [1.15 to 1.64] |
| Ethnicity, % non white | 3.8% | 5.1% | 4.7% | 6.0% | 1.26 [0.87 to 1.83] | 1.19 [0.77 to 1.85] |
| Social class  *Professional/managerial*  *other* | 64.7%  35.3% | 57.4%  42.6% | 65.5%  34.5% | 57.2%  42.8% | 0.97 [0.82 to 1.14] | 1.01 [0.84 to 1.21] |
| Suicidal thoughts, age 21 years | 12.2% | 13.2% | 44.7% | 47.4% | 5.81 [4.90 to 6.90] | 5.92 [4.86 to 7.22] |
| Suicidal plans, age 21 years | 1.5% | 1.8% | 15.2% | 18.6% | 11.7 [8.30 to 16.5] | 12.2 [8.22 to 18.3] |
| Self-harm, age 21 years | 13.1% | 13.6% | 47.3% | 49.1% | 5.97 [5.04 to 7.07] | 6.12 [5.05 to 7.42] |
| Sought professional help for self-harm or suicidal thoughts, age 21 years | 2.2% | 2.3% | 17.4% | 13.1% | 9.40 [6.98 to 12.7] | 10.2 [7.29 to 14.2] |
| Self-harm exposure (family/friend), age 16 years | 40.3% | 39.5% | 57.1% | 59.2% | 1.97 [1.66 to 2.34] | 2.22 [1.81 to 2.72] |
| Depression, age 18 years | 5.2% | 11.7% | 14.3% | 24.5% | 3.01 [2.22 to 4.10] | 2.45 [1.75 to 3.44] |
| Anxiety, age 18 years | 8.7% | 14.8% | 19.5% | 27.0% | 2.56 [1.97 to 3.31] | 2.13 [1.62 to 2.79] |
|  |  |  |  |  |  |  |
| Number of close friends, age 21 years  *3+ friends*  *0-2 friends* | 97.4%  2.6% | 96.5%  3.5% | 94.8%  5.2% | 92.7%  7.3% | 2.08 [1.43 to 3.03] | 2.17 [1.40 to 3.35] |
| Not in employment, education, or training (NEET) age 21 years | 7.9% | 10.7% | 9.2% | 14.2% | 1.17 [0.90 to 1.54] | 1.39 [1.02 to 1.89] |

*We accounted for attrition using a combination of multiple imputation and inverse probability weighting (MI/IPW). IPW is an appropriate technique given the pattern of missing data in this study (as most data was taken from a single source most participants either had complete observed or completely missing information). Multivariable Imputation by Chained Equations was used as a preliminary step to impute missing values amongst the data used in the IPW missingness model. One hundred imputed datasets were generated for responders (n=3946) and for non- responders (n=4579). Imputations were generated separately for males and females to allow for possible gender differences. The datasets were pooled and a logistic regression model was derived to predict inclusion in the final sample (n=3946). A number of variables associated with non-response were included (e.g. indicators of socioeconomic adversity, maternal psychopathology, demographics, and mental health). The Hosmer-Lemeshow test indicated that the model provided a reasonable fit to the data. The inverse of the predicted probabilities from this model were then used to weight the subsequent analyses. The largest 1% of weights were truncated to the value of the 99^th^ percentile (10.26).*

**Supplementary Table 4: Factors associated with suicide/self-harm related Internet use amongst those with a history of self-harm (n=819)**

|  | **No suicide/self-harm related Internet use**  **n=400** | **Suicide/self-harm related Internet use**  **n=419** | **OR [95% CI]** | **P value*** |
| --- | --- | --- | --- | --- |
| Gender, % female | 310 (77.5%) | 328 (78.3%) | 1.05 [0.75, 1.46] | 0.788 |
| Ethnicity, Non-white | 14 (3.7%) | 20 (5.0%) | 1.36 [0.68, 2.74] | 0.386 |
| Social class  Professional/managerial  other | 241(64.8%)  131(35.2%) | 245 (63.0%)  144 (37.0%) | 1.08 [0.80, 1.45] | 0.605 |
| Suicidal thoughts (yes vs no) | 165 (41.8%) | 267 (63.9%) | 2.46 [1.86, 3.27] | <0.001 |
| Suicidal plans (yes vs no) | 33 (8.3%) | 112 (26.9%) | 4.05 [2.67, 6.15] | <0.001 |
| Past year self-harm (yes vs no) | 88 (22.7%) | 190 (48.0%) | 3.14 [2.31, 4.28] | <0.001 |
| Sought professional help for self-harm or suicidal thoughts (yes vs no) | 67 (16.8%) | 154 (36.8%) | 2.89 [2.08, 4.01] | <0.001 |
| Self-harm exposure (family/friend) (yes vs no) | 218 (72.4) | 219 (68.2%) | 0.82 [0.58, 1.15] | 0.252 |
| Depression (yes vs no) | 33 (12.0%) | 62 (23.1%) | 2.21 [1.39, 3.50] | 0.001 |
| Anxiety (yes vs no) | 60 (21.7%) | 84 (33.2%) | 1.63 [1.11, 2.40] | 0.012 |
|  |  |  |  |  |
| Number of close friends  *3+ friends*  *0-2 friends* | 366 (94.6%)  21 (5.4%) | 385 (94.6%)  22 (5.4%) | 0.99 [0.53,1.84] | 0.990 |
| Not in employment, education, or training (NEET) (yes vs no) | 33 (8.6%) | 45 (11.1%) | 1.33 [0.83, 2.13] | 0.241 |

** Difference between those with and without suicide/self-harm related internet use in the subsample who had self-harmed*

*The number with missing data was 2 for age, 0 for gender, 39 for ethnicity, 58 for social class, 6 for suicidal thoughts, 7 for suicidal plans, 35 for past year self-harm, 0 for seeking professional help, 197 for exposure to self-harm in friend’s/family, 274 for CIS-R depression and anxiety disorder, 25 for number of close friends and 27 for NEET.*

**Supplementary Table 5: Factors associated with suicide/self-harm related Internet use amongst those without a history of self-harm (n=3, 127)**

|  | **No suicide/self-harm related Internet use**  **n=2,660** | **Suicide/self-harm related Internet use**  **n=467** | **OR [95% CI]** | **P value*** |
| --- | --- | --- | --- | --- |
| Gender, % female | 1, 523 (57.3%) | 249 (53.3%) | 0.85 [0.70, 10.4] | 0.114 |
| Ethnicity, Non-white | 96 (3.8%) | 20 (4.4%) | 1.19 [0.72, 1.94] | 0.498 |
| Social class  Professional/managerial  other | 1,618 [64.7%)  882 (35.3%) | 294 (67.7%)  140 (32.3%) | 0.87 [0.71, 1.08] | 0.223 |
| Suicidal thoughts (yes vs no) | 206 (7.8%) | 127 (27.4%) | 4.47 [3.48, 5.73] | <0.001 |
| Suicidal plans (yes vs no) | 13 (0.5%) | 22 (4.7%) | 10.1 [5.05, 20.2] | <0.001 |
| Self-harm exposure (family/friend) (yes vs no) | 720 (35.5%) | 171 (47.2%) | 1.62 [1.30, 2.03] | <0.001 |
| Depression (yes vs no) | 71 (4.2%) | 20 (6.6%) | 1.62 [0.97, 2.70] | 0.064 |
| Anxiety (yes vs no) | 112 (6.6%) | 28 (9.2%) | 1.44 [0.94, 2.23] | 0.097 |
|  |  |  |  |  |
| Number of close friends  *3+ friends*  *0-2 friends* | 2,494 (97.8%)  55 (2.2%) | 429 (94.9%)  23 (5.1%) | 2.43 [1.48, 4.00] | <0.001 |
| Not in employment, education, or training (NEET) (yes vs no) | 199 (7.8%) | 34 (7.6%) | 0.96 [0.66, 1.41] | 0.844 |

** Difference between those with and without suicide/self-harm related internet use in the subsample who had never self-harmed*

*The number with missing data was 2 for age, 0 for gender, 120 for ethnicity, 193 for social class, 15 for suicidal thoughts,15 for suicidal plans, 739 for exposure to self-harm in friend’s/family, 1,111 for CIS-R depression and anxiety disorder, 126 for number of close friends, and 134 for NEET.*
